# Sense of Control and Epigenetic Aging: The Mediating Role of Lifestyle Behaviors

**DOI:** 10.1093/geroni/igaf122.2410

**Published:** 2025-12-31

**Authors:** Kylie Schiloski, Sofia Christofi, Eric Kim, Steven Cole, Margie Lachman

**Affiliations:** Brandeis University, Waltham, Massachusetts, United States; Brandeis University, Waltham, Massachusetts, United States; The University of British Columbia, Vancouver, British Columbia, Canada; University of California, Los Angeles School of Medicine, Los Angeles, California, United States; Brandeis University, Waltham, Massachusetts, United States

## Abstract

Psychosocial factors such as purpose in life have been found to contribute to epigenetic aging and lifestyle behaviors may mediate these associations. Sense of control is another promising protective psychosocial factor that has been associated with greater engagement in protective lifestyle behaviors and better health. The present study tested whether sense of control was related to epigenetic age and whether lifestyle behaviors (diet, exercise, sleep, alcohol consumption, and smoking history) were a mediator. We predicted that individuals with a higher sense of control would engage in more protective lifestyle behaviors which would be related to a lower epigenetic age. Data were collected from two sub-study samples, the second (M2; n = 1,255) and refresher (R1; n = 863) waves of the Midlife in the United States Study (MIDUS). We tested our hypothesis with a mediation model using the PROCESS Macro in SPSS. Results showed that the relationships between sense of control and three second-generation epigenetic clocks (DunedinPACE, GrimAge, and PhenoAge) were fully mediated by lifestyle behaviors, adjusting for chronological age, sex, education, race, and study sample. In this model, sense of control was not significantly related to two first-generation epigenetic clocks (Hannum or Horvath clocks), possibly due to their higher correlations with chronological age. Taken together, these findings suggest that sense of control is a promising modifiable psychosocial variable that could be used to promote engagement in protective lifestyle behaviors potentially slowing age-related declines.

